# Renal pseudoaneurysm and arterial–pelvicalyceal fistula after partial nephrectomy managed with staged angioembolization: A case report

**DOI:** 10.1016/j.eucr.2026.103650

**Published:** 2026-10-23

**Authors:** Amirmasoud Naddafimajoumardi, Golnaz Ranjbarpazuki, Erfan Amini, Seyed Ali Momeni, Arian Yousefi Fard, Khashayar Danandeh

**Affiliations:** a Uro-Oncology Research Center, Tehran University of Medical Sciences, Tehran, Iran; b School of Medicine, Iran University of Medical Sciences, Tehran, Iran; c Department of Radiology, Lorestan University of Medical Sciences, Khorramabad, Iran; d School of Medicine, Tehran University of Medical Sciences, Tehran, Iran

**Keywords:** Partial nephrectomy, Nephron-sparing surgery, Renal pseudoaneurysm, Arteriovenous fistula

## Abstract

Delayed haemorrhage after partial nephrectomy is usually attributed to a single vascular lesion and controlled by transarterial embolization. A 62-year-old man developed massive haematuria one week after left partial nephrectomy. CT angiography showed a segmental renal pseudoaneurysm, which was embolised superselectively. Bleeding recurred within 24 hours; repeat angiography revealed a separate arterial–pelvicalyceal fistula, treated by a second embolization with durable haemostasis. Recurrence after apparently successful embolization may therefore reflect a second, unrecognised lesion rather than treatment failure. A low threshold for repeat angiography and staged, lesion-specific embolization can achieve definitive control while preserving renal parenchyma.

## 1 Introduction

Partial nephrectomy is the standard of care for many localised renal masses. Its complications range from asymptomatic and mild to symptomatic and life-threatening, and include vascular injuries such as haematoma, pseudoaneurysm, and arteriovenous fistula (AVF).^1^ Pseudoaneurysms and AVFs are the principal causes of clinically significant delayed haemorrhage, reported in roughly 1–2% of patients undergoing partial nephrectomy and somewhat more often after minimally invasive than after open surgery.^2^ Both lesions arise from iatrogenic injury to the highly vascular renal parenchyma during tumour resection and haemostatic suturing of the tumour bed: a pseudoaneurysm is, in essence, a pulsatile extraluminal collection of blood held in continuity with the arterial lumen by a fibrous capsule rather than by an intact arterial wall, and can progress to life-threatening rupture if this tenuous containment fails.^3^ Because delayed haematuria after partial nephrectomy can progress rapidly to haemodynamic instability, prompt recognition and directed imaging are essential; CT angiography is the modality of choice, offering rapid localisation of the bleeding source and delineation of the underlying vascular abnormality.^4^ Selective, and where feasible superselective, transarterial embolization has become the preferred first-line treatment, since it can achieve rapid hemostasis while limiting further loss of viable renal parenchyma.^5^

Less widely appreciated is a related but distinct injury: the arterial–pelvicalyceal (arteriocalyceal) fistula, in which an arterial branch communicates directly with the collecting system rather than with a vein. Although most often described as a primary or post-traumatic lesion, it can also arise iatrogenically, and its presence may not be evident on angiography performed at the time of treatment of a co-existing pseudoaneurysm or AVF(6). We report a patient in whom an initially successful embolization of a renal pseudoaneurysm after partial nephrectomy was followed by recurrent, more severe bleeding attributable to a separate arterial–pelvicalyceal fistula, ultimately controlled by a second targeted embolization.

## 2 Case presentation

A 62-year-old man was evaluated for a left renal tumour that had grown from 3.0 cm to 5.5 cm over the course of more than a decade of annual CT and ultrasound monitoring. He had a right pyelolithotomy 25 years prior, but no other pertinent comorbidity was noted. He had normal vital signs, no constitutional symptoms, and no haemodynamic instability at the time of the preoperative assessment. The genitourinary and abdominal examinations were uneventful; there was no palpable lump or soreness in the flanks, and there was no indication that the illness had progressed locally.

Non-contrast CT intravenous pyelography was conducted in the presence of the interval enlargement and revealed a mostly cystic mass with peripheral solid enhancing components of about 55 mm near the upper pole of the left kidney, consistent with a Bosniak grade IV lesion. The lower pole of the right kidney had two cortical cysts measuring 41 mm and 26 mm, while the left kidney had a 36 mm parapelvic cyst with multiple small cortical cysts. The renal parenchymal thickness and both pelvicalyceal systems were normal, with no calculi or hydronephrosis. Papillary renal cell carcinoma was identified by image-guided percutaneous biopsy of the upper-pole lesion, which was confirmed by immunohistochemistry (CK7, AMACR, PAX8, and vimentin positive; CD10 focally positive; GATA3 negative). In September 2025, he underwent open left partial nephrectomy of the upper-pole tumour was carried out. He was released on the fourth postoperative day after a straightforward early postoperative course.

Approximately one week after surgery he re-presented emergently with acute gross haematuria that progressed rapidly to clot retention with obstructive lower urinary tract symptoms. Haemoglobin was 6.2 g/dL. Emergency CT angiography demonstrated a pseudoaneurysm arising from a segmental branch of the left renal artery, measuring approximately 17 × 12 × 23 mm, with contrast extravasation into the collecting system; postoperative perinephric fat stranding and a small collection were also described (Fig. 1). Selective renal angiography confirmed the pseudoaneurysm (Fig. 2, arrow), and superselective transarterial embolization of the feeding branch was undertaken. Angiographic reassessment immediately afterwards confirmed occlusion of the lesion with no further opacification.

**Fig. 1:**
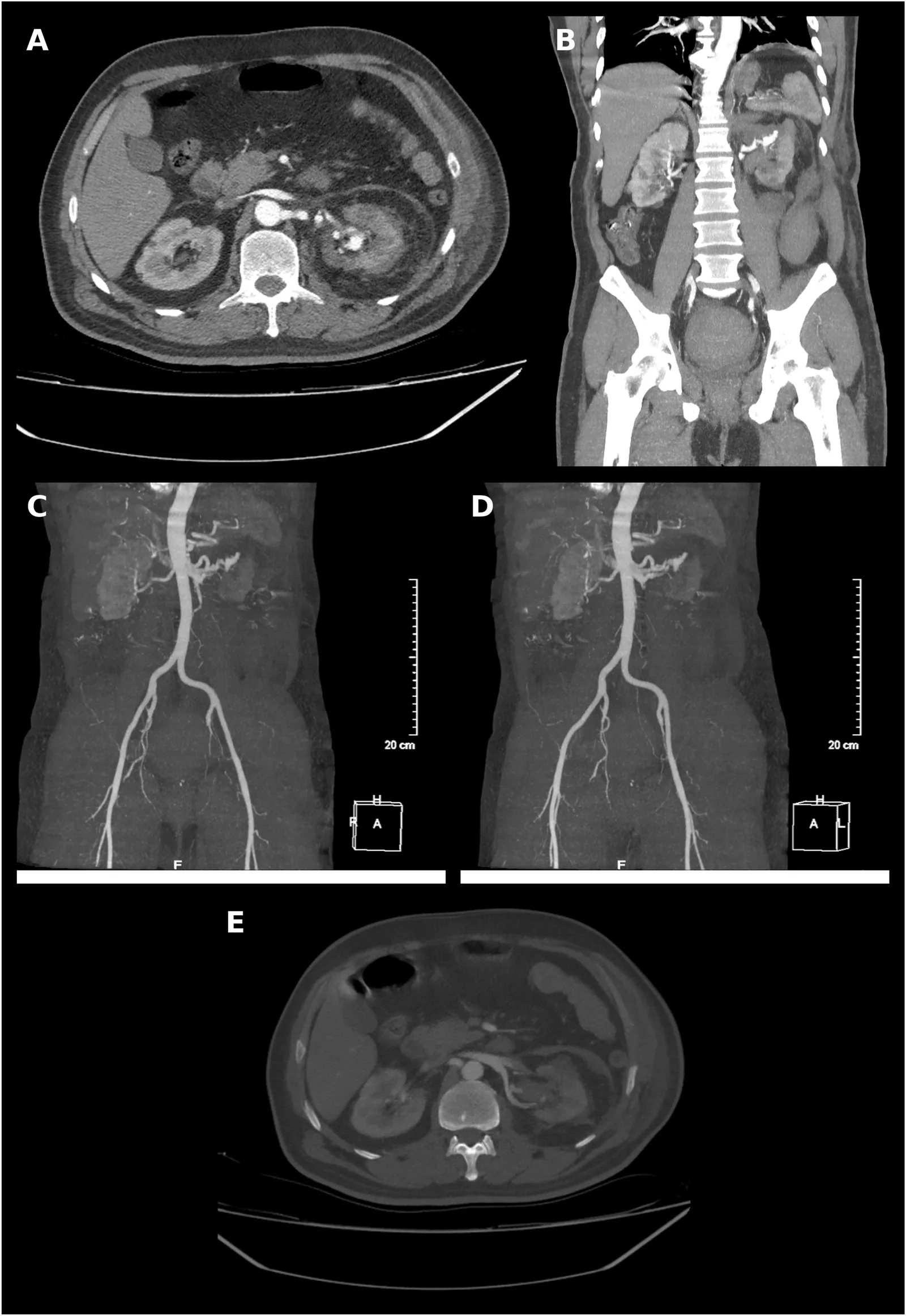
Contrast-enhanced CT angiography obtained at the first postoperative presentation with gross haematuria, approximately one week after left partial nephrectomy. (A) Axial arterial-phase image at the level of both kidneys, showing the left upper-pole resection bed with adjacent perinephric fat stranding. (B) Coronal reformation of the same acquisition, demonstrating the left kidney and the postoperative changes at the upper pole. (C) Coronal maximum-intensity-projection reconstruction of the arterial phase, showing the aorta and both renal arteries and their segmental branches. (D) Coronal maximum-intensity-projection reconstruction in a further plane, used to localise the abnormal segmental branch of the left renal artery. (E) Axial delayed (nephrographic) phase image at the same level as (A), assessed for contrast extravasation into the collecting system.

**Fig. 2:**
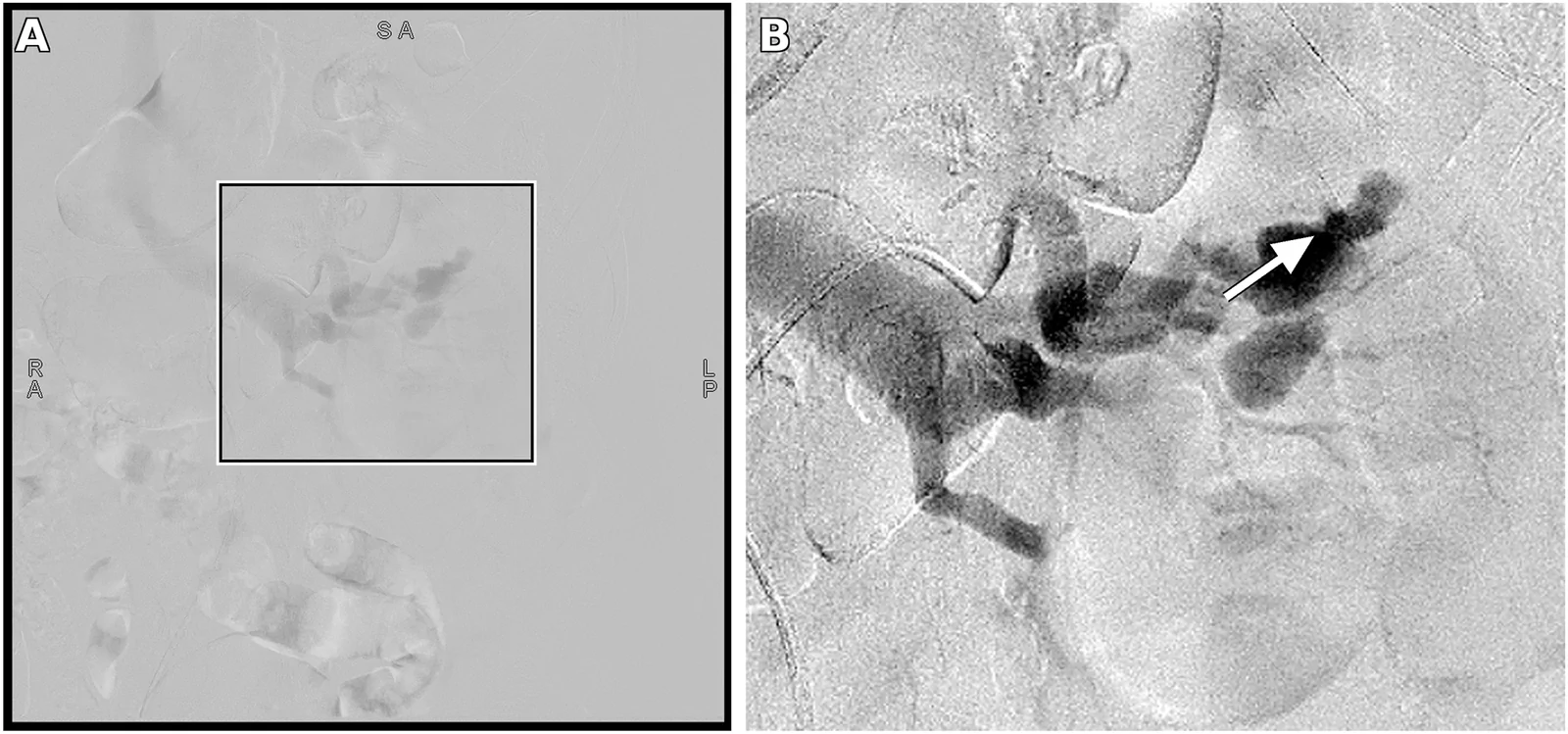
Selective digital subtraction angiography of the left renal artery at the first endovascular procedure, before embolization. (A) Full field of view; the box indicates the magnified area. (B) Magnified view of the boxed area with uniform contrast adjustment. The arrow indicates the contrast-filled pseudoaneurysm arising from a segmental branch, which was subsequently treated by superselective transarterial embolization of its feeding branch. Residual misregistration artefact from bowel gas is present.

The gross haematuria resolved for around 24 hours but subsequently reappeared, accompanied by further clot retention and a decrease in haemoglobin to 5.0 g/dL despite the administration of packed red blood cells, suggesting a persistent or new cause of haemorrhage. Recurrent cross-sectional imaging and catheter angiography revealed a further fistulous connection between a branch of the renal artery and the renal pelvis, anatomically different from the previously embolised pseudoaneurysm (Fig. 3, arrow). A further superselective embolization was executed, focusing on the supplying branch of this fistula. Angiographic re-evaluation confirmed complete occlusion of the abnormal arterial–pelvicalyceal communication, and the gross haematuria resolved promptly and did not recur. Subsequent abdominal and pelvic ultrasonography following clinical improvement revealed an unremarkable liver, spleen, pancreas, and gallbladder, with no evidence of ascites; both kidneys exhibited normal size and maintained cortical echogenicity, with no signs of hydronephrosis, calculi, or perinephric fluid accumulation, and the bladder wall appeared normal. These results align with the resolution of vascular problems; nevertheless, ultrasonography alone cannot replace confirmatory cross-sectional or angiographic assessments.

**Fig. 3:**
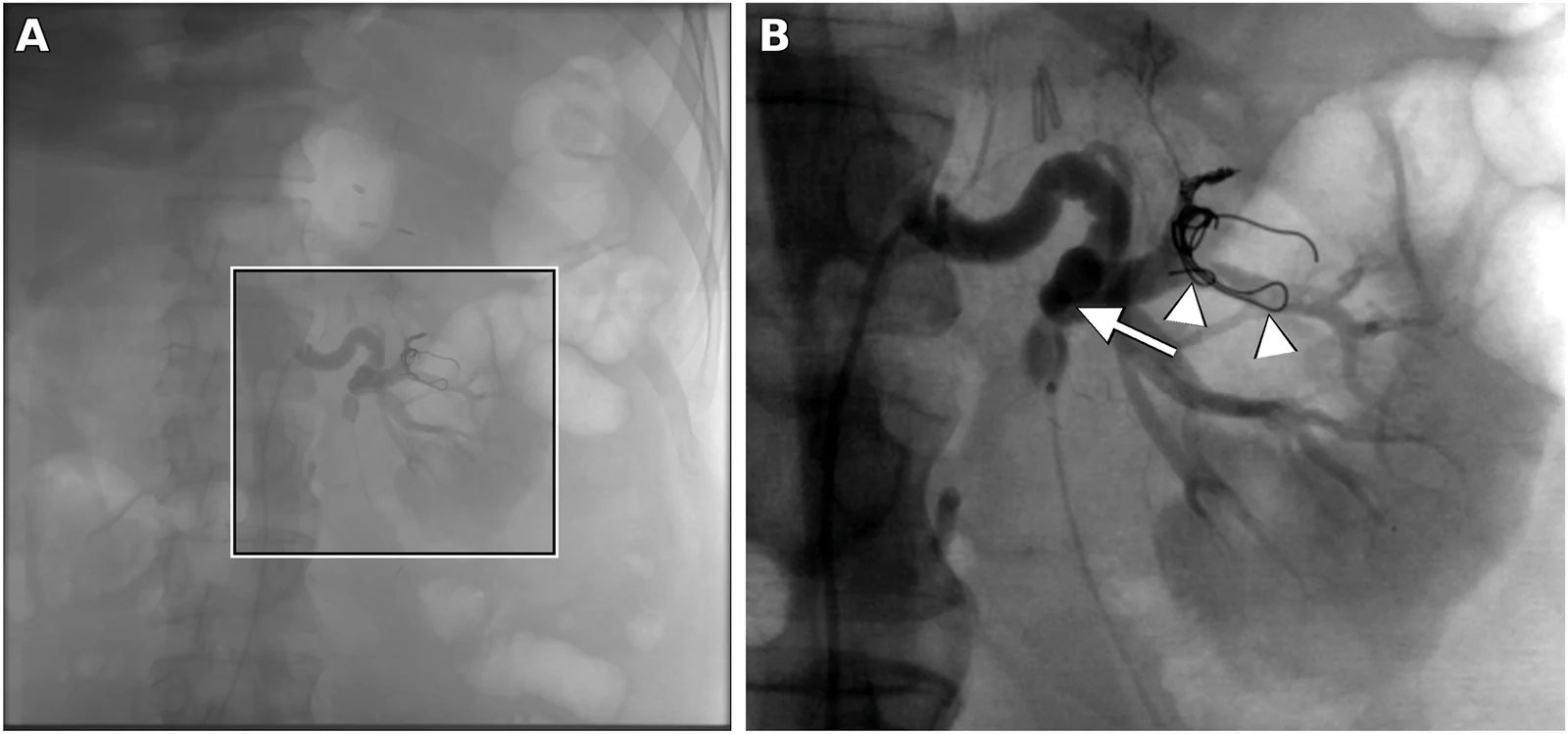
Repeat selective angiography of the left renal artery (unsubtracted image) after recurrence of gross haematuria following the first embolization. (A) Full field of view; the box indicates the magnified area. (B) Magnified view of the boxed area with uniform contrast adjustment. The arrow indicates the arterial–pelvicalyceal fistula, with abnormal opacification of the renal pelvis from a renal artery branch, anatomically separate from the previously treated pseudoaneurysm; this was the target of the second superselective embolization. Arrowheads indicate the embolization coils deployed at the first procedure.

## 3 Discussion

Delayed haemorrhage after partial nephrectomy is a recognised complication, with pseudoaneurysms and AVFs as the principal vascular aetiologies. In a systematic review pooling 5229 patients, renal artery pseudoaneurysm (RAP) was reported in 1.00% after open and 1.96% after minimally invasive partial nephrectomy (p ≤ 0.001), presenting at a mean of 14.9 days postoperatively, with gross haematuria in 87.3% of cases and a 96% success rate for percutaneous angioembolization.^2^ In one of the largest single-centre series, 39 of 1425 patients (2.7%) undergoing partial nephrectomy over ten years required embolization for a pseudoaneurysm or AVF; technical success was 100%, while clinical success, defined as no further operation or nephrectomy during follow-up, was achieved in 35 of 39 patients, four of whom required a further procedure^6^ In a large tertiary series the incidence of pseudoaneurysm was significantly lower after robot-assisted partial nephrectomy (1.0%) than after laparoscopic (3.3%) or open (3.4%) surgery.^7^ At Memorial Sloan-Kettering Cancer Center, pseudoaneurysm with or without AVF was diagnosed in 7 of 1160 patients after open (0.6%) and 8 of 301 after laparoscopic (2.7%) partial nephrectomy.^8^ Higher tumour complexity is consistently associated with these complications: in a matched case-control study, patients who developed an iatrogenic vascular lesion had significantly higher R.E.N.A.L. nephrometry scores than matched controls (8.8 ± 2.0 versus 6.5 ± 1.8, p < 0.001), although long-term renal function did not differ between groups^9^; A similar association between higher exophyticity scores and severe postoperative haemorrhage has been described in a two-centre series of 300 patients, of whom 4.3% experienced haemorrhage of Clavien grade III or higher.^10^ In our patient, sequential pseudoaneurysm and arterial–pelvicalyceal fistula became clinically apparent within the first postoperative week, underscoring the need for vigilance in the early postoperative period, particularly for tumours with documented interval growth and an upper-pole location.

Pseudoaneurysms typically result from partial arterial wall disruption, incomplete transection, needle puncture, or insufficient clamping during closure of the renal defect, leading to a contained extraluminal collection of blood^3^.^11^ AVFs arise from an abnormal communication between an artery and an adjacent vein, potentially exacerbated by intraoperative clamping or the use of thermal energy.^1^ The arterial–pelvicalyceal fistula denotes an abnormal communication between an arterial branch and the collecting system itself; when described as a primary vascular malformation, it is amenable to the same selective embolization techniques used for AVF. Notably, at least one report has observed that during embolization of an AVF the lesion may reveal itself as a frankly bleeding arteriocalyceal fistula, necessitating further embolization,^12^ a mechanism that offers a plausible explanation for our patient's course. A comparable pattern of concomitant pseudoaneurysm and AVF requiring combined embolization has been described after percutaneous nephrolithotomy.^13^ No patient-related comorbidity has been consistently identified as an independent predisposing factor.^9^ In our patient, the upper-pole location and 5.5 cm size of the tumour, together with prior contralateral pyelolithotomy, may plausibly have increased vulnerability, since interval enlargement can necessitate a more extensive resection with corresponding risk of injury to segmental vessels and adjacent calyceal infundibula.

Clinical manifestations are typically delayed, with reported median or mean times to presentation across large series ranging from approximately 12 to 15 days,^2^, ^7^, ^8^, ^14^ although individual cases have been documented from the first postoperative day to several months after surgery.^15^ Common features include gross haematuria, flank pain and, in more severe cases, clot retention, obstructive uropathy and profound anaemia; gross haematuria of any cause warrants urgent structured evaluation given its association with both benign and life-threatening pathology.^16^ Recurrent or refractory bleeding after an initially successful embolization is not unique to our case: in the Memorial Sloan-Kettering series 3 of 15 patients (20%) had persistent gross haematuria after embolization, one of whom required repeat angiography and thrombin injection of a persistent pseudoaneurysm one week after the index procedure^8^ and in a large robotic-versus-open comparative series a second embolization was required in 1 of 17 patients (5.8%).^7^ Our patient's course illustrates a related but mechanistically distinct pattern: technically successful control of one lesion followed by recurrence attributable to a second, anatomically separate injury rather than to recanalisation or incomplete treatment of the first. A post-embolization complication, upper urinary tract clot obstruction requiring endoscopic clot evacuation and ureteric stenting, has also recently been reported after otherwise successful pseudoaneurysm embolization.^17^

Prompt and accurate diagnosis is essential for managing postoperative vascular complications, with cross-sectional and catheter angiography as the cornerstones of assessment.^1^, ^4^ In our patient the initial CT angiogram demonstrated the pseudoaneurysm, while repeat imaging after recurrent bleeding revealed the separate arterial–pelvicalyceal fistula, illustrating the value of a low threshold for re-imaging when the clinical course does not follow the expected pattern. This is consistent with data indicating that colour Doppler ultrasonography, although convenient, has limited sensitivity for pseudoaneurysm (as low as 67% in one series), whereas CT angiography offers substantially higher sensitivity (98.7–100%) and specificity (90–95.1%); the same series found that early, direct angiographic intervention was associated with higher post-treatment haemoglobin, lower transfusion and antibiotic use, shorter hospitalisation and fewer re-admissions than a delayed, imaging-first pathway.^18^ Endovascular management offers minimally invasive haemostasis while preserving renal function.^14^ In a dedicated series of 25 patients with 47 pseudoaneurysms and 9 AVFs, technical success was 96% after the first attempt and 100% after a second, with clinical success of 96% and 100% respectively, and no significant change in renal function despite a mean 13.8% ± 15.1% loss of angiographically estimated kidney functional volume.^19^ Embolization has likewise been associated with a mean parenchymal volume loss of 25.2%, but no significant change in eGFR,^7^ and a matched case-control study found no significant difference in renal function at 24 months between patients requiring selective angioembolization and controls.^9^ In our case the first superselective embolization achieved immediate technical success, yet haemostatic success was only transient; a second, lesion-specific embolization was required for durable control, without complication or further renal compromise, mirroring the minority of patients in published series who require more than one procedure^6^, ^7^, ^8^, ^19^.

## 4 Conclusions

This case demonstrates that significant delayed haematuria after partial nephrectomy can, in a small number of patients, result from more than one distinct vascular injury, here a renal pseudoaneurysm and an independent arterial–pelvicalyceal fistula, rather than from a solitary lesion. Emergency CT angiography and superselective embolization achieved immediate technical control of the first lesion, but recurrence of haematuria with a further fall in haemoglobin prompted repeat imaging, which identified and allowed definitive treatment of the second, previously unrecognised fistula. Clinicians should remain alert to a second vascular abnormality when gross haematuria recurs after apparently successful post-nephrectomy embolization, since a staged, lesion-targeted endovascular approach can provide durable haemostasis while preserving renal parenchyma and avoiding open surgery. Further study of the recognition and prevention of concomitant vascular injuries during nephron-sparing surgery may help reduce the incidence of this complication and inform the timing of imaging after a first embolization.

## CRediT authorship contribution statement

**Amirmasoud Naddafimajoumardi:** Writing – original draft, Methodology, Data curation, Conceptualization. **Golnaz Ranjbarpazuki:** Writing – original draft, Data curation, Conceptualization. **Erfan Amini:** Writing – review & editing, Project administration, Data curation. **Seyed Ali Momeni:** Visualization, Supervision, Project administration. **Arian Yousefi Fard:** Writing – review & editing, Supervision. **Khashayar Danandeh:** Writing – review & editing, Writing – original draft, Supervision.

## Ethics approval and consent to participate

Not applicable.

## Consent for publication

Written informed consent for publication of this case report and any accompanying clinical images was obtained from the patient.

## Availability of data and materials

The datasets and imaging supporting the conclusions of this article are available from the corresponding author on reasonable request.

## Funding

No external funding was received for this work.

## Competing interests

The authors declare that they have no competing interests.
